# Conservative management of 900 pediatric distal radius fractures using the schede cast: a multicenter study

**DOI:** 10.1007/s00068-026-03149-w

**Published:** 2026-03-18

**Authors:** Simon Scherer, Jurek Schultz, Till Rausch, Benjamin Schoof, Laura Altmeier, Josephine Hertel, Meltem Sahin, Michael Esser, Boy Bohn, Guido Fitze, Markus Dietzel, Dirk Sommerfeldt, Justus Lieber, Kristofer Wintges

**Affiliations:** 1 Department of Pediatric Surgery and Pediatric Urology, University Children’s Hospital, University Hospital Tübingen, Hoppe-Seyler-Str. 3, 72076 Tübingen, Germany; 2 Institute of Clinical Anatomy and Cell Analysis, University of Tübingen, Elfriede-Aulhorn-Str. 8, 72076 Tübingen, Germany; 3 Clinic for Pediatric and Adolescent Surgery and Urology, Klinikum Chemnitz, Flemmingstr. 2, 09116 Chemnitz, Germany; 4 Department of Pediatric Surgery, University Medical Center Hamburg‑Eppendorf (UKE), Martinistr. 52, 20246 Hamburg, Germany; 5 Clinic and Polyclinic for Pediatric Surgery of the University Hospital Dresden, Technical University Dresden, Fetscherstr. 74, 01307 Dresden, Germany; 6 Department of Pediatric Surgery, Catholic Children’s Hospital Wilhelmsstift Hamburg, Liliencronstr. 130, 22149 Hamburg, Germany; 7 Department of Diagnostic and Interventional Radiology, University Hospital Tübingen, Hoppe-Seyler-Str. 3, 72076 Tübingen, Germany; 8 Department of Pediatric and Adolescent Traumatology, Altona Children’s Hospital, Hamburg, Bleickenallee 38, 22763 Hamburg, Germany

**Keywords:** Pediatric traumatology, Distal forearm fracture, Schede position, Conservative therapy, Plaster immobilization

## Abstract

**Background:**

Distal forearm fractures are among the most common pediatric injuries and are typically managed with cast immobilization. Volar-flexion ulnar-deviation splints (Schede casts) have been proposed to reduce displacement and prevent secondary dislocation. However, data on efficacy and safety in children remain limited. This study aimed to evaluate the clinical outcomes of Schede cast immobilization across four pediatric trauma centres over a 16-year period.

**Methods:**

We conducted a retrospective analysis of patients under 18 years with distal forearm fractures treated with Schede casts. Demographic data, fracture characteristics, initial and post-cast displacement, incidence of secondary dislocation, and complications were recorded and analyzed.

**Results:**

A total of 900 patients (mean age 9.47 years) were included. The most common mechanism of injury was sports-related trauma, and transverse metaphyseal fractures predominated. Mean initial displacement was 17.7° (± 9.4°), reduced to 5.9° (± 5.5°) after cast application and 6.9° (± 5.4°) at consolidation after a mean of 35 (± 16) days. Secondary dislocations were effectively prevented at flexion angles > 50° (*p* < 0.001). Complications were rare: tingling paresthesia occurred in 22 patients (2.4%), and prolonged movement restriction in 8 patients (0.9%). All adverse events resolved within days to weeks without long-term sequelae.

**Conclusion:**

Schede cast immobilization is a simple, safe, and effective method for retaining distal radius fractures in children. It reliably prevents secondary dislocation while maintaining low complication rates, supporting its continued use in pediatric trauma care.

## Introduction

Distal forearm fractures are the most common type of fractures in children, accounting for up to 42% of all pediatric fractures [1, 2]. Optimal management aims to achieve the best functional and anatomical outcome with minimal diagnostic and therapeutic burden [3]. The distal forearm demonstrates remarkable growth potential, contributing approximately 80% to longitudinal growth and being adjacent to a highly flexible joint, which facilitates remodeling [4]. Although dislocation limits are cited in textbooks, they are not based on prospective, randomized studies. Clinical experience suggests that fractures within these limits typically achieve complete anatomical and functional healing through growth-associated processes [5, 6].

Non-displaced or minimally displaced fractures are usually treated conservatively with cast immobilization [7]. Fractures exceeding these displacement thresholds require reduction, followed by either cast immobilization or surgical fixation with Kirschner wires, depending on stability. Surgical intervention, while effective, increases healthcare costs and carries risks including infection and neurovascular injury. Conversely, inadequate fixation or suboptimal immobilization may result in secondary dislocation, leading to functional impairment, additional surgical procedures, and prolonged treatment [8–10]. Factors contributing to secondary dislocation include fracture pattern, insufficient reduction, and poor immobilization technique. Effective prevention relies on precise anatomical reduction, correct casting technique, and supplementary stabilization methods.

The Schede cast, probably named after the German surgeon Prof. Dr. Max Schede or orthopedic surgeon Prof. Dr. Franz Schede, is one such method [11, 12]. This technique employs forced volar flexion and ulnar deviation of the wrist to counteract dorsal tilt and stabilize the fracture via ligamentotaxis [13]. It can be performed on an outpatient basis with closed reduction under analgosedation and is commonly applied directly in emergency departments [14]. Despite its potential, complications such as iatrogenic carpal tunnel syndrome, complex regional pain syndrome (CRPS), and persistent movement restrictions have been reported in adults, leading some clinicians to consider the Schede cast as outdated and to prefer surgical fixation in pediatric patients [15, 16].

Given the limited data on its use in children, this study aimed to retrospectively evaluate conservative treatment of distal forearm fractures using the Schede position. Following confirmation of feasibility in a preliminary study [17], the primary objectives were to assess overall tolerability, effectiveness in preventing secondary displacement, and the incidence of treatment-related complications. By providing comprehensive outcome data from multiple pediatric trauma centres, this study aims to support clinical decision-making and optimize conservative management strategies for this common pediatric injury.

## Method

A retrospective multicentre study was performed at four German tertiary care hospitals with a high volume of pediatric trauma patients: University Hospital Tübingen, University Medical Center Hamburg-Eppendorf, Technical University Dresden, and Catholic Children’s Hospital Wilhelmsstift Hamburg.

Eligible participants were children and adolescents aged ≤ 17 years presenting with distal forearm fractures. Fractures were included if they were classified as metaphyseal according to the AO Pediatric Comprehensive Classification of Long Bone Fractures (AO PCCF [1]; 23-M3 and 22-E1/2) or involved the radial diametaphysis as defined by the square-based radiographic model [18]. Only patients treated with immobilization in a Schede cast between 1 January 2010 and 31 December 2025 were included. Demographic characteristics, type of accident, and any abnormalities regarding peripheral circulation, movement, and sensitivity, as well as complications reported in the literature, were obtained from hospital information systems. Restricted mobility was defined as a clinically detectable limitation in active wrist or forearm range of motion compared with the contralateral side, assessed at the scheduled radiographic consolidation control. Range of motion was evaluated clinically by the treating physicians. Resolution of restricted mobility was defined as restoration of symmetric, pain-free motion without functional limitation at subsequent follow-up visits.

**Fig. 1:**
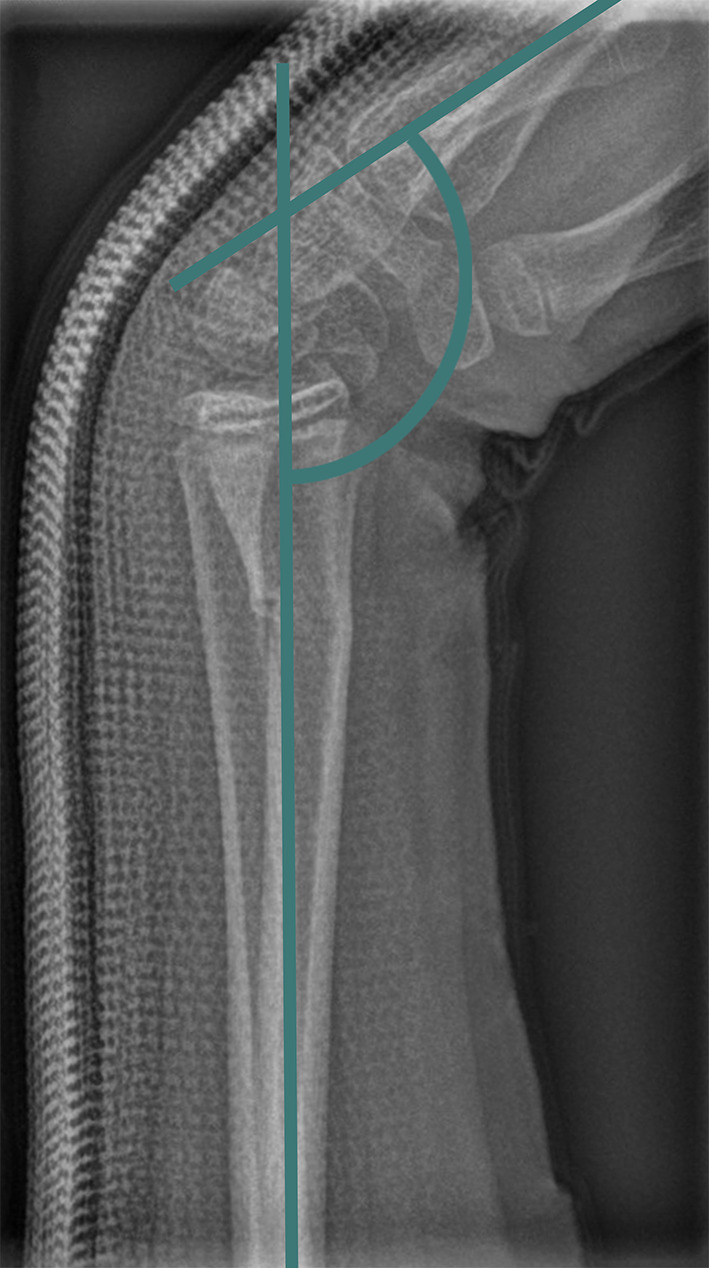
Schematic illustration of the radiological measurement of the flexion angle of the fracture position (green) in a distal radius fracture after application of the dorsal Schede cast

Displacement angles were measured from available radiographs before and after casting, with active reduction if necessary, and at consolidation [2]. The cast index (CI) was evaluated as a secondary outcome only in cases with circumferential casts. In patients treated with dorsal casts, CI was not assessed, as CI determination requires a closed (circumferential) cast geometry. CI measurements were performed at the fracture level. However, in some Schede casts, the intentional joint flexion produces a cast curvature at the fracture level that may bias measurements. In such cases, CI was determined immediately proximal to the cast flexion to ensure measurement consistency. CI values > 0.8 were considered unfavourable regarding the risk of secondary dislocation [3]. An increase in distal radial angulation of more than 6.5° was defined as clinically relevant secondary dislocation [4]. Initial displacement and cast length were also recorded. Due to the lack of standardized angle measurement protocols in the literature, the flexion angle was determined as described by Kreder et al. [2], using the axis of the radius relative to the bone axis of a representative metacarpal (Fig. 1).

Data collection and processing were performed in accordance with the latest version of the World Medical Association Declaration of Helsinki – Ethical Principles for Medical Research Involving Human Subjects. Ethics approvals were obtained from all participating centres, based on the approval of the Eberhard Karls University of Tübingen dated 13 January 2023 (026/2023BO2).

Statistical analyses were conducted using SPSS Statistics Premium 29.0.2 (IBM Corp., Armonk, NY, USA). Mean values, standard deviations, and multiple linear regression analyses were calculated. A p-value < 0.05 was considered statistically significant.

## Results

A total of 900 patients met the inclusion criteria. The left arm was affected more frequently (57.1%), and most injuries were sports-related (47%). Fractures were predominantly metaphyseal radius fractures (23r-M/3.1; 64,4%), followed by Salter-Harris II fractures (23r-E/2.1; 28.3%), diametaphyseal fractures (23r-M/3.1; 5.7%), and Salter-Harris I fractures (23r-E/1.1; 1.6%). An associated ulnar fracture was diagnosed in 462 cases (51.3%), and 50 patients (5.6%) had additional concomitant injuries. One female patient presented with a pathological fracture secondary to a juvenile bone cyst. Detailed descriptive data are summarized in Table 1.

**Table 1:** Demographic data, outcomes, and complications

Patients (n)	900
Age [years]; (mean, SD)	9.47 (± 3.2)
Sex (n, %)	
Male	619, 68.7%
Female	281, 31.3%
Affected sides (n, %)	
Left	514, 57.1%
Right	386, 42.9%
Type of accident (n, %)	
High-impact trauma	17, 1.9%
Low-impact trauma	90, 10%
Sports accident	423, 47%
Minor trauma	362, 40.2%
Other/Unknown	8, 0.9%
Fracture type (n, %)	
Salter-Harris I fracture	14, 1.6%
Salter-Harris II fracture	255, 28.3%
Metaphyseal fracture	580, 64.4%
Diametaphyseal fracture	51, 5.7%
Dislocation before Schede P.T. [°]; (mean, SD)	17.7 (± 9.4)
Dislocation after Schede P.T. [°]; (mean, SD)	5.9 (± 5.5)
Dislocation at consolidation P.T. [°]; (mean, SD)	6.9 (± 6.4)
Immobilization in (n, %)	
Upper arm cast	723, 80%
Forearm cast	177, 20%
Angle of flexion [°]; (mean, SD)	50.9 (± 14.1)
Flexion angle of the respective cast [°]; (mean, SD)	
Upper arm cast	52.5 (± 18.5)
Forearm cast	44.8 (± 14.9)
CI; (mean, SD)	0.86 (± 0.1)
<0.8 (n)	153
Pathological fractures (n, %)	1, 0.1 %
Change of procedure	27, 3 %
Transient tingling paresthesia	22, 2.4 %
Temporary movement restrictions	8, 0.9 %
Cast removal in days (mean, SD)	34 (± 10)
Return to sports in days (mean, SD)	53 (± 17)

**Fig. 2:**
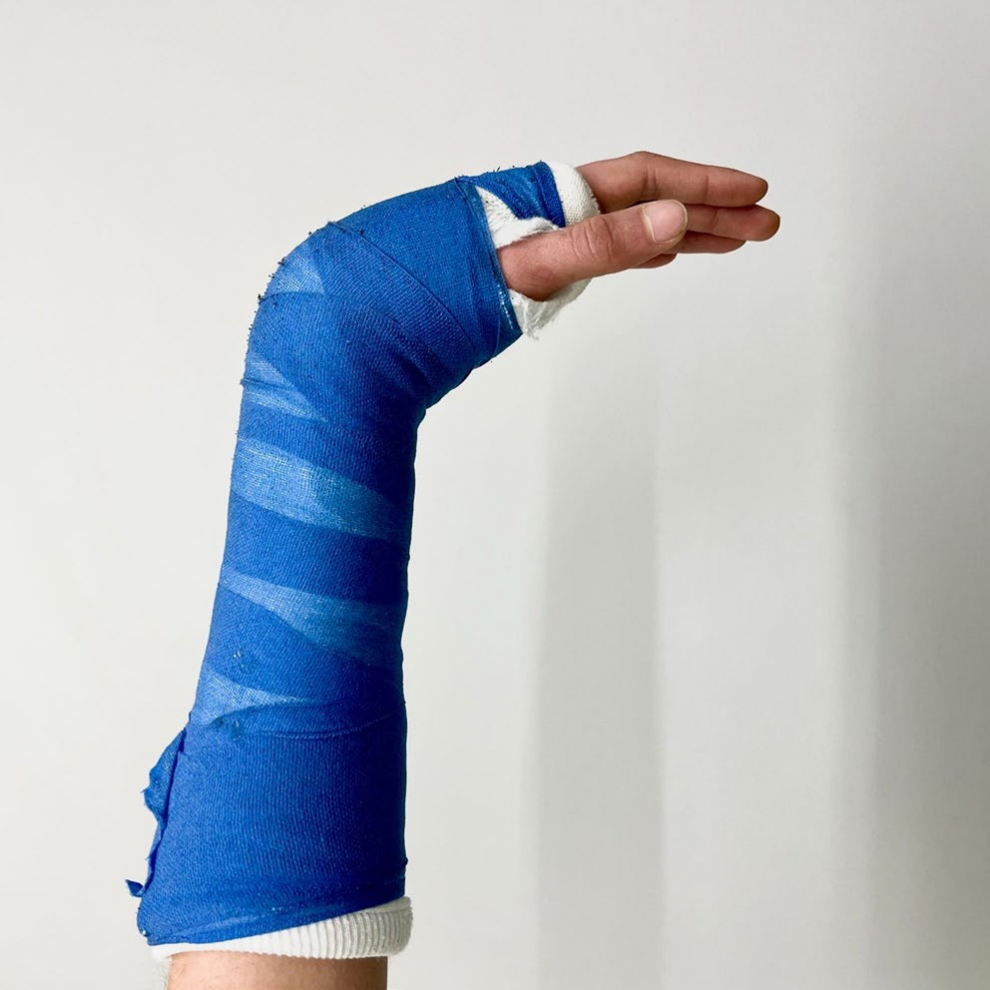
Dorsal forearm cast in Schede position at the time of consolidation

**Fig. 3:**
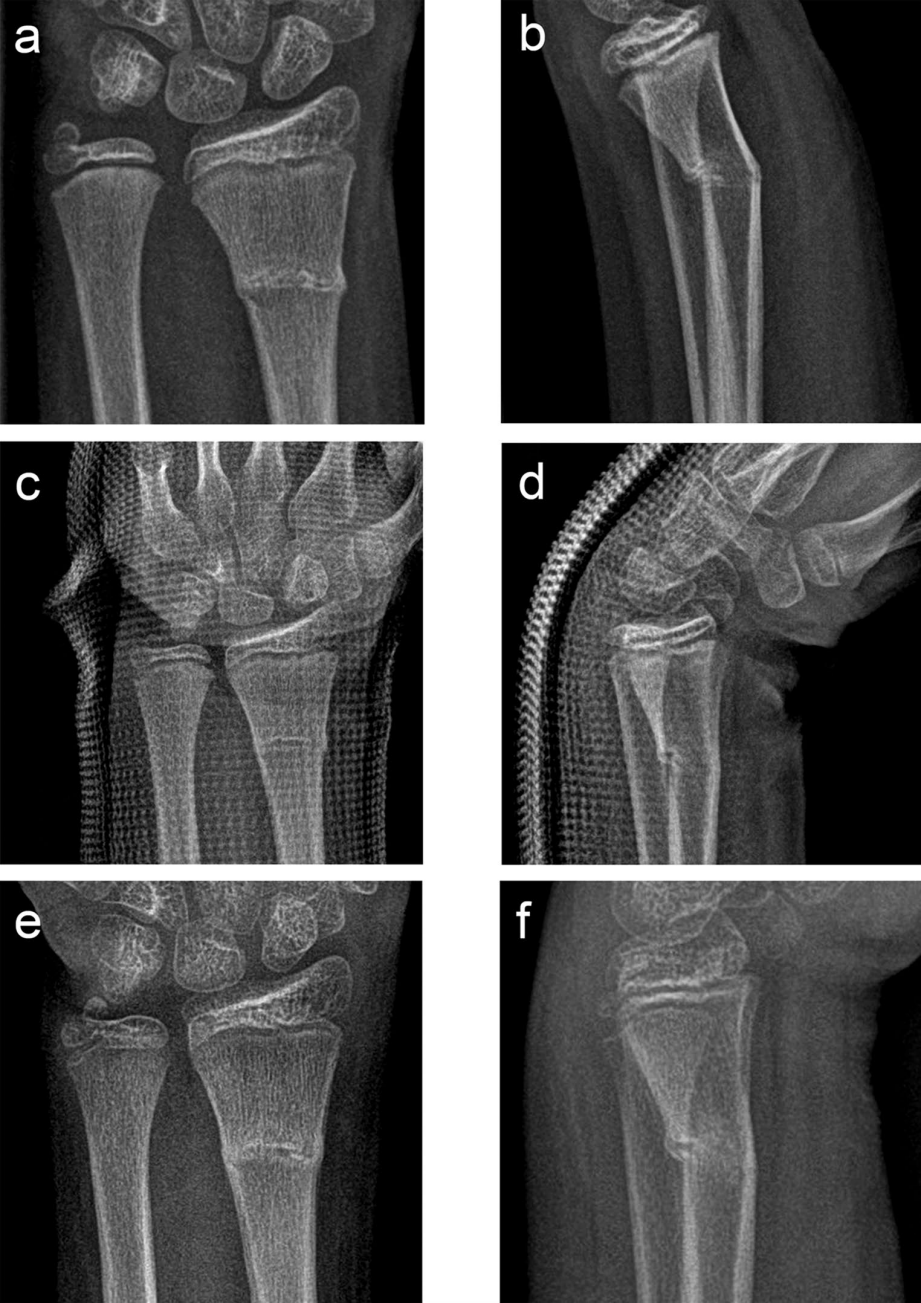
Ten-year-old female patient with a distal metaphyseal transverse fracture of the radius and an avulsion fracture of the ulnar styloid process on the left side following a fall from a swing (**a**, **b**). Radiographic alignment control on day 7 after reduction and cast application in a Schede cast under analgosedation (**c**, **d**). Radiographic consolidation control on day 27 followed by clearance for spontaneous mobilization (**e**, **f**). Clearance for sports activities was granted on day 50 after an unremarkable final clinical examination (no radiographic follow-up)

All patients were initially retained in a Schede cast (Fig. 2). Mean initial displacement was 17.2°, reduced to 5.5° after casting, with or without active reduction, and measured 6.2° at consolidation. During treatment, an average of eight (± 3) radiographs were obtained: for diagnosis verification/before casting, after reduction and Schede cast application, at one-week follow-up, and at consolidation, each in two planes (exemplary course in Fig. 3). Post-reduction control was performed using a mobile C-arm in 48% of cases.

An upper arm cast was applied in 80% of patients, with a mean flexion angle of 52.5° (± 18.5°), significantly higher than forearm casts (44.8° ± 14.9°; *p* < 0.001). The mean CI was 0.86 (± 0.1), with forearm casts showing a significantly lower CI than upper arm casts (0.81 ± 0.1 vs. 0.9 ± 0.3; *p* < 0.001). The mean observation period was 63 days (± 80), with cast removal after 34 (± 10) days and return to sports at 53 (± 17) days.

In 27 cases (3%), treatment was changed to reduction and Kirschner wire fixation due to secondary dislocation. Paresthesia occurred in 22 cases (2.4%), resolving within one week. Five cases involved the median nerve, two the ulnar nerve, and one the radial nerve; the remainder presented non-specific symptoms, mostly tingling in multiple fingers. Prolonged restricted mobility was observed in eight patients (0.9%), which resolved completely with physiotherapy no later than six weeks after the planned consolidation control. Table 2 summarizes factors associated with procedural change and secondary dislocation. Significant predictors included a lower flexion angle and a poorer reduction outcome in the Schede cast (9.0° vs. 5.8° and 41.9° vs. 51.2°, respectively; both *p* = 0.001). Forearm cast immobilization was more common in patients with subsequent procedural change, but this difference was not statistically significant. Neither pre-casting displacement nor the CI was identified as a significant predictor. Secondary dislocations exceeding 6.5° were analyzed separately, as current guidelines do not provide specific thresholds for defining minimal deterioration. This cutoff was therefore used to identify clinically relevant secondary displacement at an early stage. Significant factors associated with secondary dislocation included a lower flexion angle (45.2° vs. 52.7°; *p* < 0.001) and insufficient fracture reduction, reflected by greater residual displacement after casting (8.6° vs. 5.6°; *p* = 0.036) and at consolidation (15.4° vs. 5.7°; *p* < 0.001). Secondary dislocation was more common in younger children (8.1 vs. 9.6 years; *p* = 0.01). CI, initial displacement, and the presence of an associated ulnar fracture did not differ significantly between groups.

**Table 2:** Group comparison

Procedural changes (n = 27)	Total	Yes, changed	No	p-value
Angle of flexion [°]	50.9 (± 14.1)	41.9 (± 3.7)	51.2 (± 3.3)	< 0.001
Age [years]	9.47 (± 3.2)	10.3 (± 3.7)	9.4 (± 3.3)	0.087
Immobilization in (n, %^a^)				
Upper arm cast	723 (80.3 %)	19 (2.1 %)	704 (78.2 %)	0.639
Forearm cast	177 (19.7 %)	8 (0.9 %)	169 (18.8 %)	
Cast index	0.86 (± 0.1)	0.9 (± 0.2)	0.86 (± 0.3)	0.279
Dislocation before Schede P.T. [°]	17.7 (± 9.4)	16.8 (± 9.1)	17.7 (± 10)	0.602
Dislocation after Schede P.T. [°]	5.9 (± 5.5)	9 (± 7.3)	5.8 (± 5.5)	0.001
Additional ulna fracture (n, %^a^)				
Yes	462 (51.3 %)	13 (1.4 %)	449 (50 %)	0.836
No	438 (48.7 %)	14 (1.5 %)	424 (47.1 %)	
**Secondary dislocation > 6.5° (n = 112)**	**Total**	**Yes, dislocated**	**No**	**p-value**
Angle of flexion [°]	50.9 (± 14.1)	45.2 (± 17.7)	52.7 (± 17.8)	< 0.001
Age [years]	9.47 (± 3.2)	8.1 (± 3.5)	9.6 (± 3.2)	0.01
Immobilization in (n, %^a^)				
Upper arm cast	757	91 (10.1 %)	666 (74 %)	0.965
Forearm cast	143	21 (2.3 %)	122 (13.6 %)	
Cast index	0.86 (± 0.1)	0.9 (± 0.3)	0.86 (± 0.3)	0.275
Dislocation before Schede P.T. [°]	17.7 (± 9.4)	17.3 (± 8.7)	17.7 (± 10.1)	0.974
Dislocation after Schede P.T. [°]	5.9 (± 5.5)	8.6 (± 7.1)	5.6 (± 5.3)	0.036
Dislocation at consolidation P.T. [°]	6.9 (± 6.4)	15.4 (± 8.2)	5.7 (± 5.2)	< 0.001
Additional ulna fracture (n, %^a^)				
Yes	455	48 (5.4 %)	407 (45.2%)	0.188
No	445	64 (7.1 %)	381 (42.3%)	

Table 3 summarize transient paresthesia and prolonged movement restrictions. Higher age was associated with both complications (11.4 vs. 9.4 years; *p* = 0.016 and 12.1 vs. 9.4 years; *p* = 0.015). Both occurred more frequently in boys and were more common in upper arm casts compared with forearm casts. Flexion angle and duration of immobilization did not influence complication rates.

**Table 3:** Complications

Paresthesia (*n* = 22)	Total	Yes	No	*p*-value
Angle of flexion [°]	50.9 (± 14.1)	48.5 (± 11.3)	50.9 (± 18.1)	0.31
Age [years]	9.47 (± 3.2)	11.4 (± 3)	9.4 (± 3.3)	0.16
Immobilization in (n, %) Upper arm cast Forearm cast	723 (80.3%) 177 (19.7%)	18 (2%) 4 (0.4%)	705 (78.3%) 173 (19.2%)	0.467
Period of immobilization [days]	35 (± 16)	36.2 (± 14.1)	35 (± 20.4)	0.841
**Restriction of movement (n = 8)**	**Total**	**Yes**	**No**	**p-value**
Angle of flexion [°]	50.9 (± 14.1)	43.5 (± 14.6)	50.9 (± 17.9)	0.105
Age [years]	9.47 (± 3.2)	12.1 (± 2.9)	9.4 (± 3.3)	0.015
Immobilization in (n, %)				0.323
Upper arm cast	723 (80.3 %)	5 (0.56 %)	718 (79.8 %)	
Forearm cast	177 (19.7 %)	3 (0.33 %)	174 (19.3 %)	
Period of immobilization [days]	35 (± 16)	41.3 (± 9.8)	34.9 (± 20.3)	0.204

## Discussion

This study demonstrates that treatment of children with distal forearm fractures in the Schede position is uncomplicated, effective, and safe. Given the high incidence of these fractures in children, treatment efficiency is of great importance. Many of these injuries can be managed conservatively with cast immobilization, with or without prior closed reduction, taking advantage of the excellent growth potential of the distal forearm.

The optimal joint position during immobilization remains a subject of debate, with various techniques described in the literature to prevent secondary fracture displacement and to achieve an anatomically correct and functional joint position at the end of treatment [5, 6]. In this study, the Schede cast immobilization technique was analyzed, as ligamentotaxis is intended to minimize mechanical stress on the fracture and reduce the risk of redislocation. Despite the recognized benefits of exploiting pediatric remodeling potential, concerns about retention therapy in the Schede cast have persisted for nearly a century. These concerns are based largely on adult data and contrast with positive clinical experience in pediatric practice.

The first reported case of iatrogenic carpal tunnel syndrome (CTS) following Schede cast immobilization of a distal radius fracture in adults was described by Abbott and Saunders [7], with subsequent studies attributing this complication to increased carpal tunnel pressure with wrist flexion [8, 9]. Although CTS is generally considered rare in children, it has been reported predominantly in association with distal radius pathology, particularly following trauma [19]. however, non-traumatic cases have also been described. In the present series, all cases of neurological irregularities were fully reversible, even in the presence of transient median nerve sensory disturbances. No impairment of two-point discrimination was observed, and there was no indication for surgical decompression. This favorable course may be explained by the higher tolerance of children and adolescents to elevated compartment pressures [10], which may allow more effective compensation for cast-induced increases in carpal tunnel pressure [8].

Temporary tingling paresthesia was observed in 2.4% of our patients and resolved spontaneously within days or after simple measures such as analgesia, cooling, or cast adjustment. The symptoms were generally non-dermatomal, likely reflecting tight casting or post-traumatic swelling. Notably, these paresthesias occurred more frequently in older children and in boys, possibly due to less flexible fascial sheaths in male adolescents [20].

Prolonged movement restrictions were rare and resolved completely with physiotherapy. As expected, these limitations were more common in older children and in upper arm casts, likely reflecting decreased activity and slower recovery during puberty [13, 14]. Compared to adult studies reporting secondary dislocation rates of up to 39% [21], the rate of procedural change in our cohort was remarkably low at 2.9%, despite a higher proportion of radiographically detected secondary dislocations (12.4%). This discrepancy can be explained by two main factors. First, secondary dislocation was defined as a deterioration of ≥ 6.5° on follow-up radiographs, a deliberately low threshold chosen to enable early detection of subtle loss of reduction and to ensure comparability across participating centers with heterogeneous treatment protocols. Second, the distal forearm in children exhibits a high remodeling potential, allowing for considerable tolerance of mild secondary displacement without the need for surgical intervention. Patients with secondary dislocation were younger and more frequently treated with forearm casts, which were associated with lower mean flexion angles compared with upper arm casts. These findings support previous reports emphasizing the importance of adequate wrist flexion, with at least 45° recommended for Salter–Harris I and II fractures [22]. Our data suggest that this principle is also applicable to unstable meta- and diametaphyseal fractures, provided a flexion angle of at least 50° is maintained. CI values were generally high in our cohort (0.86 and 0.9), and measurement was challenging in certain cast types: dorsal casts do not allow valid CI determination, and in Schede casts, intentional joint flexion produces a cast curvature at the fracture level that can bias measurements. Previous studies have suggested that CI values above 0.8 are associated with secondary dislocation. In our study, however, nearly all measured CI exceeded this threshold, limiting variability and preventing meaningful analysis. Accordingly, no association between CI and secondary dislocation was observed, suggesting that CI may have a limited predictive role in this context [3, 23]. Concomitant ulnar fractures were not a statistically significant risk factor, although insufficient reduction remained a major predictor of secondary displacement [24]. In our cohort, conversion to closed reduction and Kirschner wire fixation was required in only 27 cases. This rate is substantially lower than those reported in previous studies, in which secondary procedural changes after an initial conservative treatment approach were described in up to 45% of cases [25, 26]. Limitations of this study include its retrospective and observational design without a standardized protocol. Due to the lack of a validated method for measuring ulnar abduction, only flexion was measured, although the Schede position includes both flexion and ulnar deviation. Nevertheless, the large sample size reflects real-world pediatric practice and demonstrates that Schede cast retention is a low-complication, conservative treatment for distal forearm fractures in children and adolescents, often avoiding surgical intervention. Compared with Kirschner wire fixation, Schede cast treatment is cost-effective, outpatient-friendly, and minimally invasive. Prospective studies, including randomized controlled trials, are needed to further validate these findings.

## Conclusion

Retention treatment of pediatric distal radius fractures in the Schede position is straightforward, safe, and effective. It can be performed in the emergency department under sedation and provides efficient conservative management, often obviating the need for surgery.

## Data Availability

The data that support the findings of this study contain sensitive patient information and are therefore not publicly available. Data can be made available upon reasonable request to the corresponding author, subject to approval by the relevant ethics committee and adherence to applicable privacy regulations.

## Abbreviations

CI: Cast Index
CRPS: Complex Regional Pain Syndrome
CTS: Carpal Tunnel Syndrome
